# Dynamic analysis of the interactions between Si/SiO_2_ quantum dots and biomolecules for improving applications based on nano-bio interfaces

**DOI:** 10.1038/s41598-018-23621-x

**Published:** 2018-03-27

**Authors:** Miruna Silvia Stan, Ludmila Otilia Cinteza, Livia Petrescu, Maria Alexandra Mernea, Octavian Calborean, Dan Florin Mihailescu, Cornelia Sima, Anca Dinischiotu

**Affiliations:** 1 University of Bucharest, Faculty of Biology, Department of Biochemistry and Molecular Biology, 91-95 Splaiul Independentei, 050095 Bucharest, Romania; 2 University of Bucharest, Faculty of Chemistry, 4-12 Bd. Regina Elisabeta, 030018 Bucharest, Romania; 3 University of Bucharest, Faculty of Biology, Department of Anatomy, Animal Physiology and Biophysics, 91-95 Splaiul Independentei, 050095 Bucharest, Romania; 4 National Institute for Laser, Plasma and Radiation Physics, 409 Atomistilor, 077125 Bucharest-Magurele, Romania

## Abstract

Due to their outstanding properties, quantum dots (QDs) received a growing interest in the biomedical field, but it is of major importance to investigate and to understand their interaction with the biomolecules. We examined the stability of silicon QDs and the time evolution of QDs – protein corona formation in various biological media (bovine serum albumin, cell culture medium without or supplemented with 10% fetal bovine serum-FBS). Changes in the secondary structure of BSA were also investigated over time. Hydrodynamic size and zeta potential measurements showed an evolution in time indicating the nanoparticle-protein interaction. The protein corona formation was also dependent on time, albumin adsorption reaching the peak level after 1 hour. The silicon QDs adsorbed an important amount of FBS proteins from the first 5 minutes of incubation that was maintained for the next 8 hours, and diminished afterwards. Under protein-free conditions the QDs induced cell membrane damage in a time-dependent manner, however the presence of serum proteins attenuated their hemolytic activity and maintained the integrity of phosphatidylcholine layer. This study provides useful insights regarding the dynamics of BSA adsorption and interaction of silicon QDs with proteins and lipids, in order to understand the role of QDs biocorona.

## Introduction

During the last decade, semiconductor quantum dots (QDs) have received a tremendous interest in the nanotechnology field, due to their unique advantages provided by optical and electrical properties^1^. The increased number of reports on QDs biological use in diagnostic, drug delivery and cellular imaging applications^2,3^ has triggered various questions concerning their interactions with cells in the physiological environment. In addition, the toxicological evaluation of nanoparticles performed, in order to show the adverse effects, have reported different conclusions for the same type of material. Consequently, the reported results became controversial and inconsistent, due to an insufficient nanoparticles characterization in the tested biological systems.

It is widely accepted that upon entering the biological media, the surface of nanoparticles will interact with the fluid’s components, providing a new biological identity for nanoparticles^4^. Different biological molecules, mainly proteins and also certain lipids, nucleic acids and even biological metabolites, compete in order to adsorb on the surface of nanoparticles. Initially, the abundant proteins rapidly and reversible bind nanoparticles and form the “soft corona”. Furthermore, these proteins, with fast exchange rates, will be replaced by long-term binding proteins with higher affinity, but less motile (“hard corona”)^5^. The formation of protein corona is a competitive process, its structure and composition depending on three main factors: nanoparticles (composition, size, surface curvature, surface charge, surface chemistry, and hydrophobicity/hydrophilicity), physiological environment (composition, protein concentration, transport through cell compartments, etc.) and time of exposure^6^. In response, the new biological identity can modify the nanoparticles size, zeta potential and colloidal stability and can influence subsequent physiological responses, such as interaction with cell membranes, cellular uptake, transport, accumulation and cytotoxicity^7^.

The interface between particles and cell membrane comprises a dynamic series of interactions that are mediated by the protein corona, and at the same time can be a valuable target for therapeutic drug delivery if an endocytosis or direct penetration is possible without any damage. Nanoparticles not only induce changes in the conformation and biological function^8,9^ of proteins adsorbed on their surface, but can also significantly modify the structure of membrane proteins and phospholipids upon their interaction^10^. Therefore, it is of great importance to follow the progressive events triggered during exposure to nanoparticle at different time points to obtain a relevant analysis.

In the current study, we aimed to provide a deeper understanding of nanoparticles behavior in biological systems by evaluating the changes of QDs’ physicochemical parameters in various biological media over time and by investigating the time evolution of silicon-based QDs_protein corona formation and its effect on cell membrane integrity, aspects important for the design of strategies able to prevent QDs’ toxicity.

## Results

### Characterization of Si/SiO_2_ QDs stability over time

**Figure 1:**
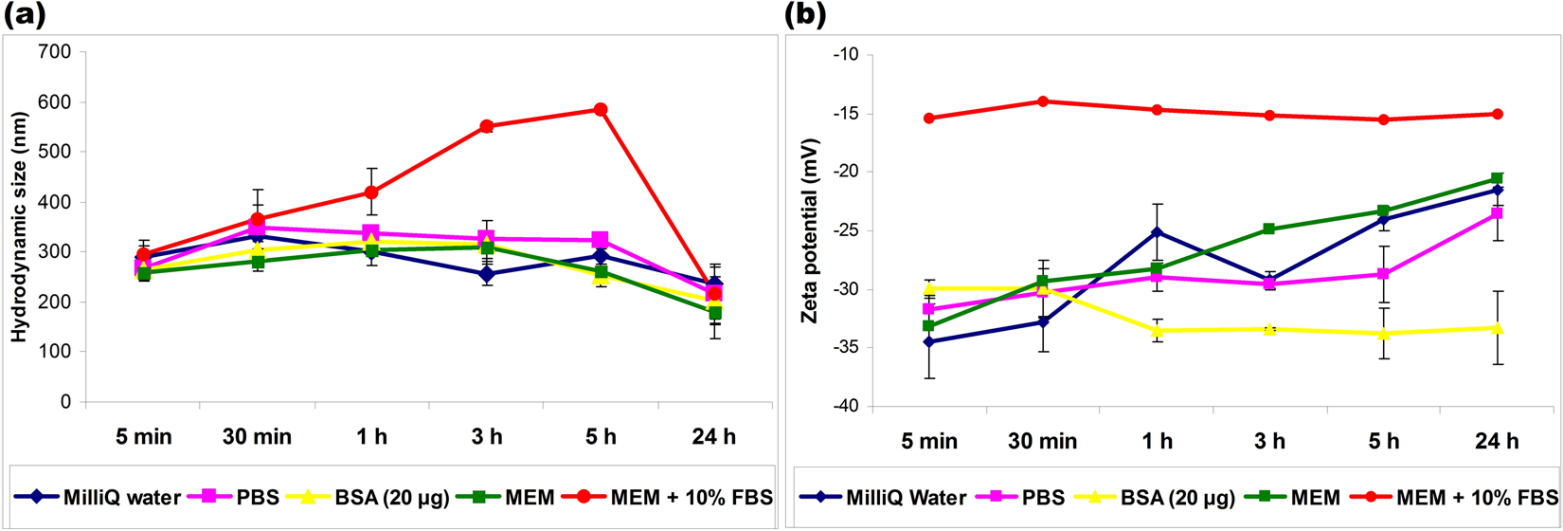
Time course analysis of hydrodynamic size (**a**) and zeta potential (**b**) of Si/SiO_2_ QDs in different media. Results are expressed mean ± SD (n = 3).

The hydrodynamic size (Fig. 1a) and zeta potential (Fig. 1b) of Si/SiO_2_ QDs were assessed to provide their physicochemical behavior in various biological media. These nanoparticles exhibit intrinsic aggregation due to the method of synthesis (i.e. laser ablation without any stabilizer) and thus, the hydrodynamic size was between 250 and 300 nm depending on the suspension media, significantly higher in comparison with the size of primary size crystallites (i.e. 6–8 nm). The evolution of QDs size in water or PBS was almost similar and did not register significant changes over 24 h. The presence of BSA induced an increase of hydrodynamic size in the first hour of incubation, due to a relatively fast adsorption of protein onto the surface of QDs. During the next 23 h, the hydrodynamic diameter of particles slightly decreases, as a result of re-arrangement of protein chains on porous surface of the Si/SiO_2_ aggregates and possibly due to the changes in secondary structure. Furthermore, this pattern of size evolution in BSA solution was very close to the one obtained in MEM. The presence of FBS induced a progressive increase of QDs size measured by dynamic light scattering (DLS), with a maximum level after 5 h that significantly dropped after 24 h at a level similar with the other media. The complex composition of MEM supplemented with 10% FBS, containing many proteins in small quantities, leads to a competitive adsorption, with a longer period of time needed to reach the equilibrium in the formation of biocorona.

Freshly dispersed Si/SiO_2_ QDs in water displayed a zeta potential of about −35 mV, indicating a good stability in aqueous solution. A small decrease to almost −25 mV was recorded after one hour, and ζ-potential remained almost unchanged up to 24 h of incubation. The presence of BSA accompanied by the BSA adsorption onto Si/SiO_2_ QDs resulted in a minor modification of the zeta potential, with values between −35 mV and −30 mV for the whole period investigated. This relative constant level of zeta potential indicated the QDs stability over time. At neutral pH, BSA adsorption is mostly due to the van der Waals interactions and the zeta potential of nanoparticles covered with biocorona exhibits small changes even at a high concentration of protein in solution^11^. The incubation with MEM + 10% FBS yielded a value of −15 mV for the zeta potential of QDs after the first 5 min, significantly higher than the values recorded in all other media (which were below −30 mV) and was maintained almost unchanged during the whole incubation period. The ζ-potential recorded for Si/SiO_2_ QDs in this type of medium was stabilized very rapid to a value close to that measured for the proteins in FBS solution alone, as it was reported by other groups^12,13^. It is presumable that other proteins in FBS than BSA, with faster adsorption dynamics, and also positively charged amino acids (such as L-glutamine from MEM composition) could be responsible for the changes in zeta potential in the first 5 min, compared to the longer period of time requested for the achievement of equilibrium in the case of corona formation in BSA solution.

This rapid increase in the case of MEM supplemented with FBS in comparison with water, PBS, BSA and MEM suggested that serum proteins affected QDs stability due to a fast and continuous interaction with nanoparticles. Similar results were revealed for different types of nanoparticles^12–14^, showing that a high amount of proteins can rapidly shift the zeta potential of nanoparticles which achieved almost a saturation level of adsorption from the first minutes of incubation.

### Analysis of Si/SiO_2_ QDs protein corona

**Figure 2:**
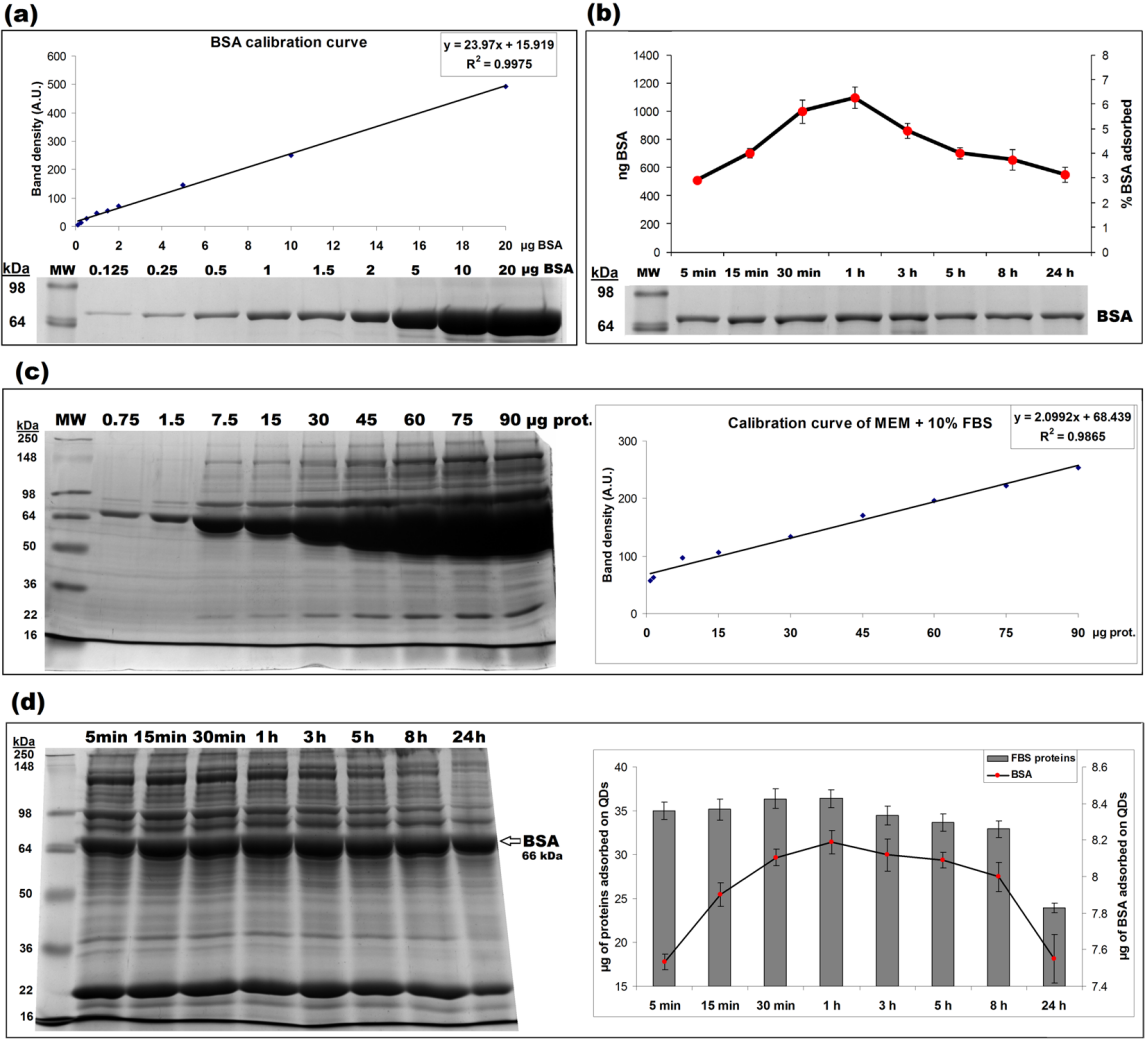
Time evolution of Si/SiO_2_ QDs protein corona in different media. (**a**) Representation of a BSA calibration curve obtained in the range of 0–20 µg by SDS-PAGE. (**b**) QDs were incubated with 20 µg/ml BSA and the amount of adsorbed BSA was calculated as ng after extrapolating the band intensity on the BSA standard curve and expressed also as % of the initial amount of suspended BSA. (**c**) Representation of a MEM + 10% FBS calibration curve obtained for protein levels in the range of 0.75–90 µg by SDS-PAGE. (**d**) QDs were incubated with MEM supplemented with 10% FBS and the total amount of adsorbed proteins and the level of adsorbed BSA were quantified based on the MEM + 10% FBS calibration curve and expressed as µg. Results are expressed mean ± SD (n = 3).

To get deeper insights into the analysis of Si/SiO_2_ QDs biocorona, the protein adsorption dynamics was investigated using sodium dodecyl sulfate-polyacrylamide gel electrophoresis (SDS-PAGE) (Fig. 2). The nanoparticles were incubated with 20 µg/ml BSA (purity higher than 98% and a molecular weight of ~66 kDa according to manufacturer’s datasheet) and in MEM with 10% FBS, and the quantification of the amount of adsorbed protein was performed using corresponding calibration curves (Fig. 2a and c, respectively). The QDs adsorbed almost 3% of the total amount of BSA even from the first 5 min, the level increasing to a maximum during the first hour (Fig. 2b). Afterwards, the process of desorption occurred, resulting in a decrease of BSA level, which was rapidly until 5 h, and much slower during the next hours. The time evolution of BSA level adsorbed on QDs, estimated by SDS-PAGE, was in agreement with the DLS measurements of QDs size in BSA solution (Fig. 1a), confirming the BSA adsorption/desorption process. Moreover, the QDs efficiently develop a corona of bovine serum proteins during incubation with FBS as shown in Fig. 2d. Right after the first 5 min of incubation, the QDs adsorbed a significant amount of FBS proteins, which clearly modified the zeta potential of nanoparticles compared to values measured for QDs in water, as represented in Fig. 1b. Although the total level of proteins slightly elevated during the first 30 min, the quantification of adsorbed BSA (the abundant protein of FBS) showed a progressive increase during the first hour (Fig. 2b). After a small decrease in the amount of bound proteins assessed at 3, 5 and 8 h, a considerable desorption was detected at 24 h, validating the QDs hydrodynamic size reduction between 5 h and 24 h of incubation. According to Bradford’s method, BSA amount represented 44% of all protein content, which was in the range provided by the manufactured for the FBS composition. The results shown in Table 1 revealed that the percentage of BSA adsorbed on QDs was lower than half of the initial ratio (18.6%), indicating that other proteins, represented in smaller amounts in FBS, had a higher affinity than BSA for QDs. Also, it is very important to highlight that QDs were able to form a protein corona composed of multiple proteins, adsorbed in a more homogenous proportion compared to FBS pattern, as it was noticed by SDS-PAGE (Fig. 2c-left versus Fig. 2d-left).

**Table 1:** Dynamic adsorption of proteins by Si/SiO_2_ QDs in different media expressed in percentages normalized to the initial protein content.

Media	Protein	Control		Time of incubation with QDs							
		Initial protein amount	% of all protein content	5 min	15 min	30 min	1 h	3 h	5 h	8 h	24 h
BSA	BSA adsorbed on QDs (%)	20 µg	100%	2.91%	4.02%	5.72%	6.27%	4.93%	4.02%	3.74%	3.15%
MEM + 10% FBS	Protein adsorbed (% of all protein content)	6.75 mg	100%	0.60%	0.61%	0.63%	0.62%	0.59%	0.58%	0.57%	0.41%
	BSA adsorbed on QDs (% of initial BSA amount)	3 mg	100%	0.253%	0.266%	0.273%	0.276%	0.273%	0.272%	0.269%	0.254%
	BSA adsorbed on QDs (% of all proteins adsorbed)	3 mg	44%	18.60%	19.08%	19.18%	19.51%	20.54%	20.80%	20.84%	27.17%

### Si/SiO_2_ QDs influence the secondary structure of BSA

**Figure 3:**
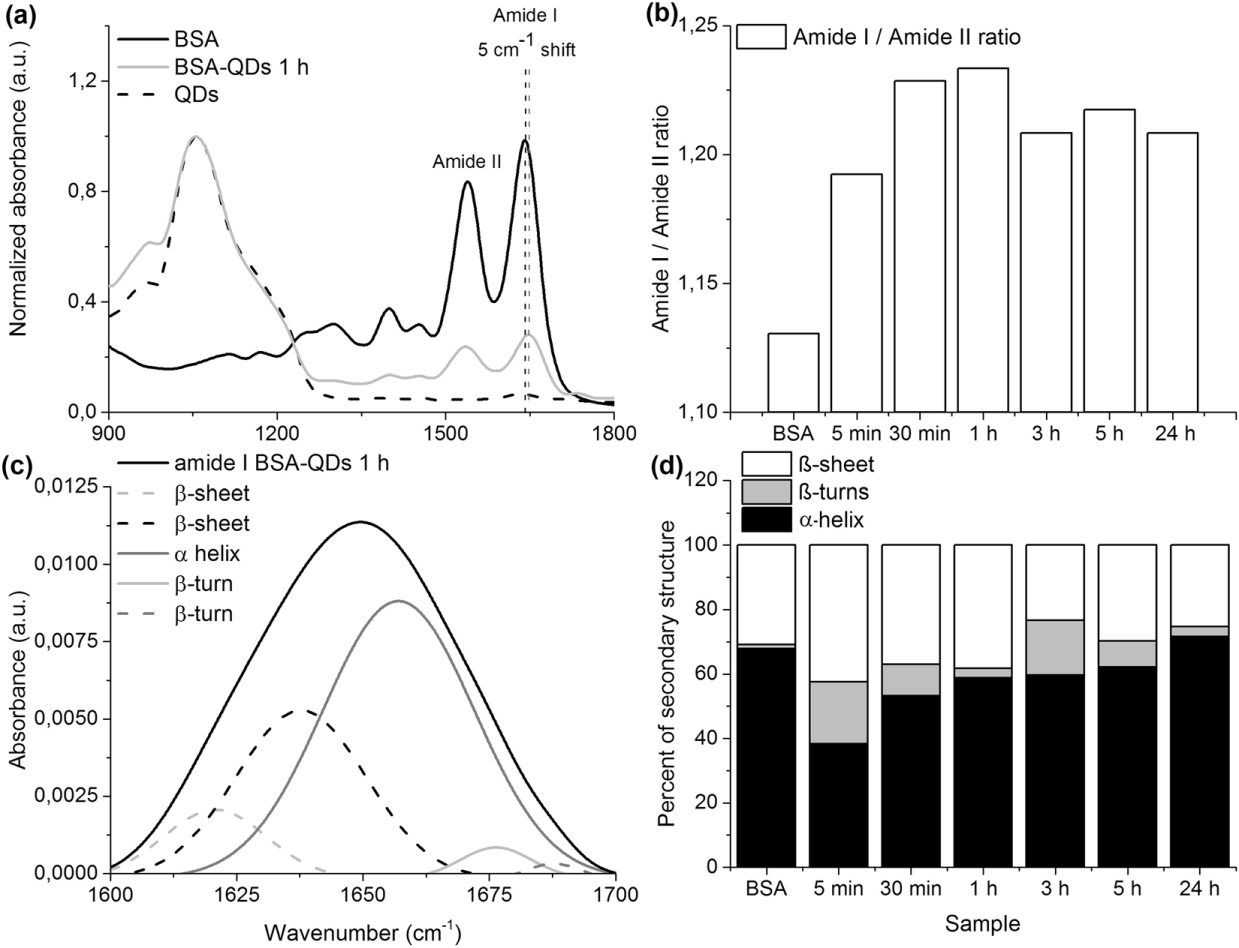
Si/SiO_2_ QDs influence the secondary structure of BSA. **(a**) The IR spectra of BSA, BSA-QDs 1 h and QDs normalized to 1. The amide I and amide II bands from the spectra of BSA and BSA-QDs 1 h are labeled on the figure. The central frequency of BSA and BSA-QDs 1 h amide I bands are marked with dotted lines and the frequency shift is also labeled on the figure. (**b**) The amide I – amide II ratios calculated for the spectra of BSA and of BSA-QDs samples. (**c**) The deconvolution of amide I band of BSA-QDs 1 h was performed on the baseline corrected spectra passing through the ordinate at 1700 cm^−1^ and 1600 cm^−1^. The location of constituent bands was identified by analyzing the minima on their second derivatives spectra. The association of resulting Gaussians with secondary structures is given in the plot legend. (**d**) The secondary structure percents of BSA and BSA-QDs samples.

The IR spectrum of QDs shows a distinct absorption band centered at ~1057 cm^−1^, due to Si-O-Si stretching vibration as previously described for silicon QDs^15^. As it can be seen in Fig. 3a, the absorption band specific to QDs is present on BSA-QDs spectra, and is absent from BSA spectrum. In the case of BSA and BSA-QDs spectra we monitored the characteristics of amide I (1600–1700 cm^−1^) and amide II (1510–1580 cm^−1^). The presence of QDs and the increase of incubation time shifted the central frequency of these bands (Fig. 3a) and changed the amide I/amide II ratio (Fig. 3b). A small shift of the peak from ~1644 to ~1649 cm^−1^, due to the perturbation of the signal of C=O stretch, is related to the interaction between the BSA chain and the surface of Si/SiO_2_ QDs, in agreement with other data reported on BSA adsorption on SiO_2_ surfaces^16^. The shift to a higher frequency suggests an increase in β-sheet and turn structures^17,18^, which are consistent with the analysis of FTIR spectra described below. The plot represented in Fig. 3b shows that BSA had the lowest amide I/amide II ratio which increased for BSA-QDs samples at 5 min, 30 min and 1 h, and afterwards decreased, but having a value still higher than in the case of BSA-QDs at 5 min.

In order to establish the protein structural changes induced by QDs binding, we performed the deconvolution of amide I bands for BSA samples. The deconvolution of amide I band for BSA-QDs 1 h sample and the structural assignment of resulting Gaussians are revealed in Fig. 3c. The secondary structure composition of samples was calculated based on the integrated area of the Gaussians and the results (%) were presented in Fig. 3d. Lyophilized BSA comprises mostly α-helices and smaller percents of β-sheets and β-turns. Upon the interaction with QDs, α → β transitions were noticed in the BSA secondary structure. Although the most dramatic changes in the BSA secondary structure, represented by 30% loss of helicity and elevated β-turns content, were observed after the first 5 min of incubation, the percent of α-helix increased over the time until 24 h when the secondary structure resembled with the one of native BSA.

### Protein corona attenuates the hemolytic activity of Si/SiO_2_ QDs

**Figure 4:**
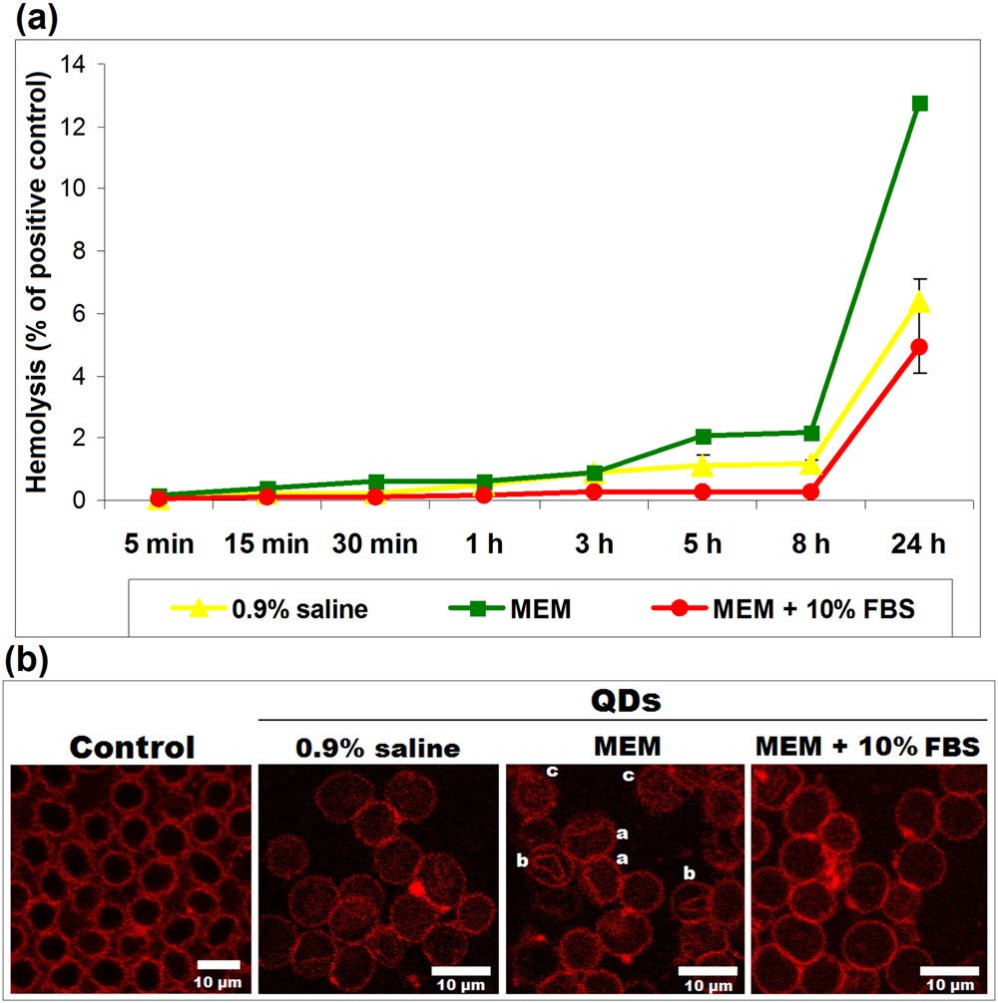
(**a**) Hemolytic activity of Si/SiO_2_ QDs in various biological media. (**b**) Representative confocal microscopy images with hypericin staining of fresh RBCs in 0.9% saline without QDs (control) and RBCs incubated for 24 h with QDs suspended in various biological media. Note altered RBCs after incubation with QDs suspended in MEM: degmacytes - a, stomatocytes - b and echinocytes - c.

A hemolysis assay was performed during the 24 h of incubation (Fig. 4a) in order to investigate the effect induced by Si/SiO_2_ QDs on the integrity of red blood cells (RBCs) membranes, According to the recommendations of ASTM F 756-00^19^. The very low hemolysis percents (below 2%), obtained in the first 8 h of incubation, indicate that QDs are non-hemolytic materials regardless of the biological medium used for obtaining the suspension. However, the lowest level of hemoglobin released during exposure was noticed in the case of QDs suspended in FBS-supplemented medium (only 4.9% of positive control after 24 h versus 12.8% for QDs in MEM) proving the protective effect of protein corona. The hemolytic activity of QDs in MEM was very well correlated with the morphological changes of RBC membrane, shown by confocal microscopy (Fig. 4b). Besides erythrocytes with normal shape, altered RBCs (degmacytes, stomatocytes and echinocytes) were also observed after incubation with QDs suspended in MEM, confirming their damaging effect on cell membrane shape and integrity after 24 h. This higher hemolytic activity induced by QDs in MEM after 24 h, almost double compared to QDs suspended in 0.9% saline, could be due to the fact that MEM is not a proper medium to preserve RBCs at good viability for a longer period of time, as it was also suggested by other researchers who have worked on fish erythrocytes and noticed a decrease of growth rate after 8 h in MEM compared to other media^20^. Although the control values were subtracted from the QDs samples values, it should be mentioned that the optical density (OD) obtained for MEM without QDs was 0.285 compared to 0.140 for 0.9% saline. Most probably the presence of QDs in MEM in the absence of serum proteins induced changes in the tonicity of solution, triggering an osmotic shock followed by changes in membrane morphology and hemolysis. Thus, the RBCs become more susceptible to a direct interaction with QDs which can alter the structure of membrane’s components.

### Protein corona influences the interaction between Si/SiO_2_ QDs and a lipid monolayer

**Figure 5:**
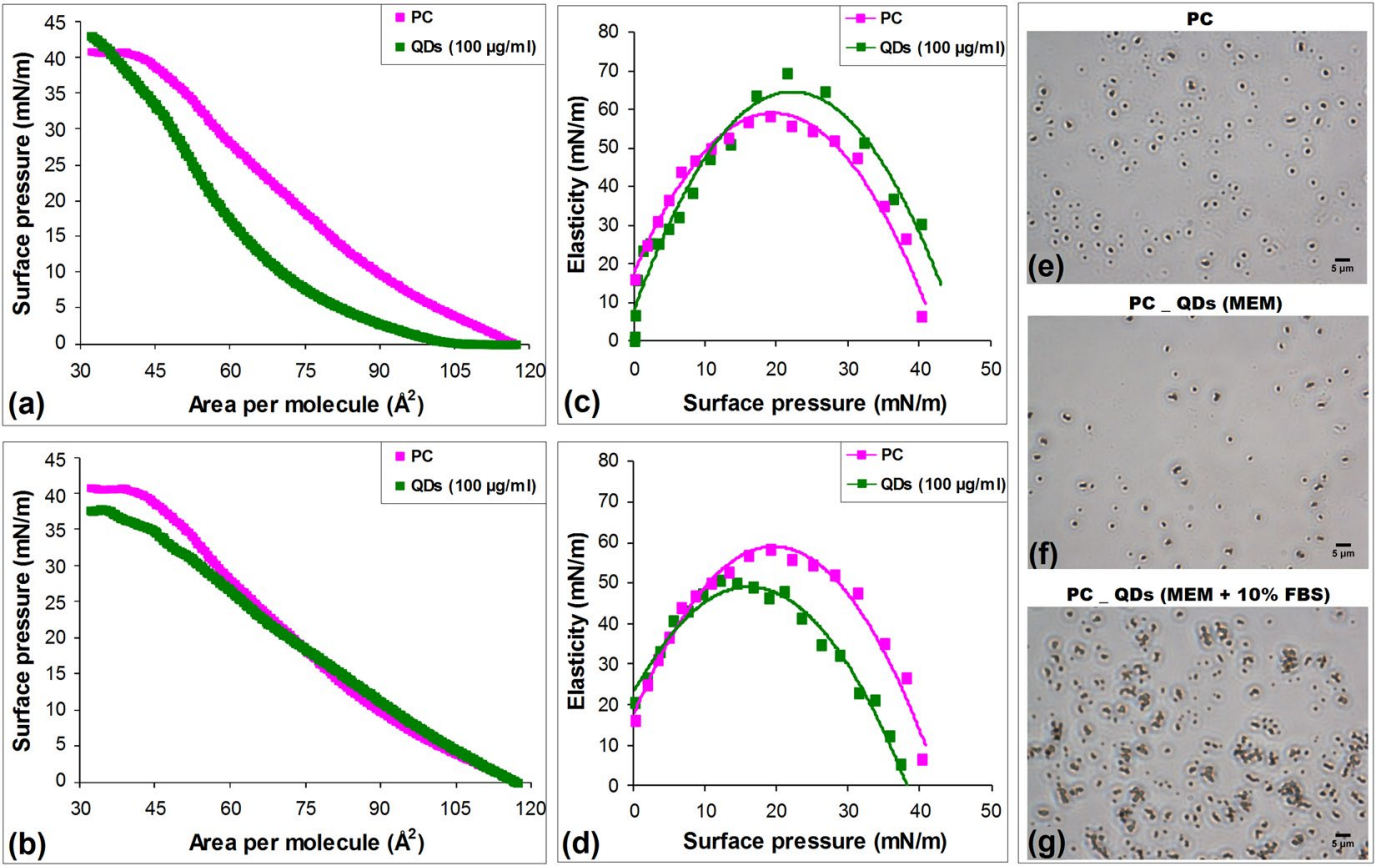
Analysis of Si/SiO_2_ QDs – lipid monolayer interactions. Surface pressure-area isotherms of PC were measured in the presence of 100 µg/ml QDs suspended in MEM (**a**) and MEM supplemented with 10% FBS (**b**). Elasticity (*Cs*^−1^) of PC monolayers in the presence of QDs suspended in MEM (**c**) and MEM supplemented with 10% FBS (**d**) were calculated and represented as a function of the surface pressure (π). Representative phase-contrast images showing the morphology of PC monolayer alone (**e**) or in the presence of 100 µg/ml QDs suspended in MEM (**f**) and MEM with 10% FBS (**g**), collected at a surface pressure of 30 mN/m.

The understanding of Si/SiO_2_ QDs potential effects on biological membranes involved the analysis of QDs – phospholipid layer interactions using the Langmuir-Blodgett trough. The addition of QDs prepared in MEM induced a shift of the whole curve to smaller areas per molecule compared with PC standard alone, and a change in the slope of the plateau to higher pressures, indicating that the lipid molecules were not squeezed out until 42 mN/m (Fig. 5a). In contrast, the QDs in MEM supplemented with 10% FBS shifted the isotherms to higher areas per molecule until a pressure of 18 mN/m was reached, followed by a transition to lower pressures compared with the pure lipid was observed (Fig. 5b) suggesting the incorporation of QDs covered with serum proteins into the PC monolayer.

The interaction of QDs with the PC monolayer could affect its rigidity. Thus, the surface compressional modulus (*Cs*^−1^) was calculated from the surface pressure isotherms data and plotted in Fig. 5c,d. The elasticity increased for QDs in MEM (Fig. 5c), but diminished in the presence of QDs with FBS (Fig. 5d). This indicated that serum proteins adsorbed on the surface of QDs facilitated their contact with the lipids, the monolayer becoming more rigid. By contrast, the QDs in MEM triggered a monolayer expansion by altering the phospholipids’ arrangement and increasing their fluidity. The images of phase-contrast microscopy confirmed these changes induced by QDs on the morphology of PC. Figure 5f clearly shows a reduction in the number of phospholipids triggered by QDs in comparison with the pure monolayer (Fig. 5e). These results explain the increased monolayer elasticity (Fig. 5c) that required higher surface pressures in order to collapse (Fig. 5a). QDs incorporation into the PC monolayer as inferred by isotherms measurements was shown in Fig. 5f, the protein corona being responsible for this ‘sticker’ effect.

## Discussion

QDs exhibit numerous potential advantages in nanomedicine as therapeutic and diagnostic tools, but the interpretation of the toxicological evaluation must include a fundamental understanding of nanoparticles behavior in biological systems. The motivation of our study was to investigate the dynamic nano-bio interactions that are of primary interest for nanotoxicology and for designing nano-devices for next-generation therapy.

The Si/SiO_2_ QDs were able to develop efficiently a protein corona, either during incubation with a single protein (BSA) (Fig. 2a), or in a complex biological medium (MEM with 10% FBS) (Fig. 2b). Due its great availability and multiple uses in various assays, BSA represents the best choice to investigate the time-course interactions between QDs and a single protein. The significant amount of adsorbed BSA measured by SDS-PAGE (Fig. 2b) after the first 5 min of incubation revealed its rapid interaction with Si/SiO_2_ QDs, which might be explained by the high-affinity of this protein towards the QDs surface. The electrostatic interaction can play an important role in BSA adsorption, as nanoparticles obtained by laser ablation, without being electrostatically stabilized, promote BSA adsorption, due to increased changes in entropy^18^. However, it was reported that BSA adsorbed on the surface of various types of nanoparticles, regardless of their size and surface charge^21^. The progressive adsorption of BSA during the first hour, followed by desorption, influenced the physicochemical characteristics upon interaction with biological medium, causing a similar change of the hydrodynamic size (Fig. 1a). Also, the zeta potential remains below −30 mV and relatively constant for Si/SiO_2_ QDs in BSA solution (Fig. 1b), suggesting that from all biological media tested in the present study this solution can maintain the best colloidal stability of these nanoparticles.

Furthermore, the incubation with MEM supplemented with 10% FBS showed a protein corona formed rapidly (within the first minutes), that changed over time only in terms of the amount of bound proteins, and not in composition (Fig. 2d), this finding correlating with a recent study on silica nanoparticles^22^. Hence, these data could suggest that the Vroman effect^23^ is not applicable for different types of nanoparticles, including Si/SiO_2_ QDs. The kinetics of serum protein adsorption validated the fast formation of protein corona, and the pattern of total protein adsorbed was compared with the amount of adsorbed BSA, the dominant protein in FBS (Fig. 2d). The profiles of adsorbed BSA (as a pure protein and in FBS) showed the same tendency of progressive increase during the first hour of incubation. During the next hours, the pattern of desorption was different, the BSA following the behavior of the other serum proteins. Therefore, the behavior of a certain protein in terms of binding affinity and adsorption/desorption kinetic rate is critically influenced not only by the nanoparticle properties, but also by the medium composition^6^.

A significant difference was noticed regarding the percent of BSA adsorbed on QDs from all bound protein amount after the incubation in MEM + 10% FBS compared to the BSA abundancy in FBS standard (Table 1 and Fig. 2c,d). Most probably, other proteins present in FBS composition, such as apolipoproteins A-I and A-II, α-glycoprotein and hemoglobin fetal subunit β, exhibit higher dynamics of adsorption/desorption than BSA, resulting in a rapid increase in the amount of adsorbed proteins, the level being maintained during the first 8 h of incubation. Afterwards, a general desorption of all proteins was recorded after 24 h, due to the slow desorption of BSA and other proteins. The different abundancy in proteins observed for the corona of Si/SiO_2_ QDs compared to the serum composition was mostly influenced by the physicochemical properties of these nanoparticles, as it was shown that each given nanomaterial has an “unique” protein corona that can be a predictor for its cytotoxicity^24,25^.

The significant increase in hydrodynamic size, during the first 5 h (Fig. 1a), supports both protein corona formation and competitive absorption and desorption in order to achieve the equilibrium. Also, a higher protein concentration in the biological environment can trigger higher thickness of corona and important changes in the relative abundancy of adsorbed proteins. Clearly, this affected also the colloidal stability of QDs, as shown by zeta potential value (Fig. 1b), the loss of stability being responsible for the tendency of these QDs to agglomerate in the presence of FBS. Proteins-mediated agglomeration was reported also for others nanoparticles^26,27^.

As revealed by FTIR spectroscopy, the binding of BSA on the surface of QDs can have a major impact on protein secondary structure and conformation (Fig. 3). The frequency and intensity of bands corresponding to amide I (arising from C=O stretching vibration of peptide linkage) and amide II (derived mainly from N-H bending vibrations) provided useful information regarding the BSA structure and stability on various nanoparticle surfaces^18,28–30^. The shift of amide I peak position (with an average value of 5 cm^−1^ after the incubation with QDs) and the increased ratio of amide I/amide II compared to native BSA clearly indicated the BSA adsorption on the Si/SiO_2_ QDs surface and the subsequent changes in protein local conformation. Notably, the spectral changes demonstrated a significant decrease of α-helix content and an increase in β-sheet amount in the secondary structure of adsorbed BSA after 5 min of incubation with QDs. According to a previous study^31^, this could be an indicator of protein aggregation on the surface of QDs, due to their hydrophobic surface. This property of Si/SiO_2_ QDs could explain also the rapid adsorption of BSA, within 5 min, and the increase in N-H bending vibrations (Fig. 3b). The α → β rearrangements in the protein secondary structure could be due to the fact that QDs bound to the amino acid residues of BSA, disrupting the hydrogen bonding networks, which could favor the formation of extended conformations, such as β-type elements. However, this structural destabilization was not perpetuated over the time, as the content in α-helices and β-sheets became similar to native BSA after 24 h of incubation. BSA was able to regain its secondary structure, and the transition between conformations could have an important impact on the function of this protein. The conformational changes, including protein aggregation, may appear as a part of the recognition system, in order to promote the excretion/degradation of foreign elements, such as QDs.

The first interaction between nanoparticles and cells takes place at the cell membrane and is critical for further nanoparticle-induced cytotoxic effects. Human RBC membrane contains approximately equal amounts of proteins and lipids and is considered an appropriate model to study nanoparticle-cell interactions. The protein corona diminished the hemolytic activity of Si/SiO_2_ QDs RBCs compared to MEM without FBS (Fig. 4a), displaying an improved hemocompatibility. However, although the hemolysis for this serum-free medium was low (below 15% of positive control), the confocal microscopy revealed the occurrence of various RBCs with abnormal shape (Fig. 4b), indicating damage of the plasma membrane. This finding is in agreement with a recent study on silica nanoparticles^32^. The presence of degmacytes and echinocytes could be related to the oxidative stress induced by QDs in MEM on human RBCs^33^. Similar morphological changes were observed in drug-related hemolysis^34^ and after incubation with platinum nanoparticles^35^, respectively.

**Figure 6:**
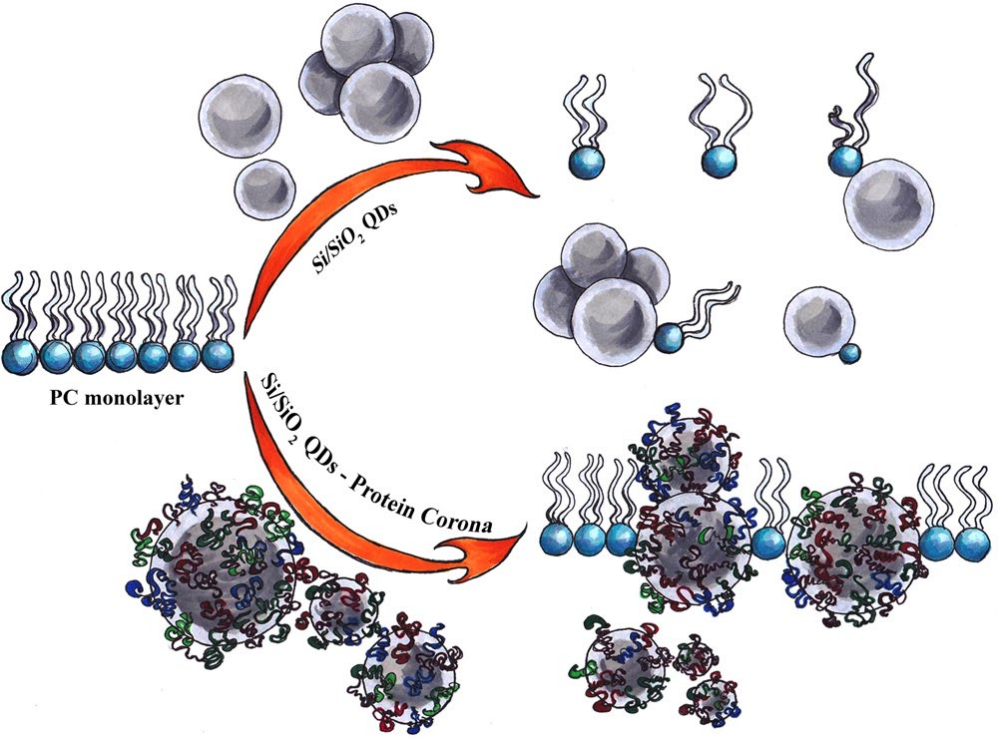
The main effects induced by Si/SiO_2_ QDs on the phospholipid monolayer. In the absence of FBS, the interaction with QDs caused the expulsion of PC molecules and their oxidative injury which affected the structure of phospholipids and disturbed their organization creating holes in the monolayer. By contrast, QDs covered with protein corona strongly interacted with PC and were incorporated among the lipids, increasing the monolayer’s stiffness.

Protein adsorption contributes significantly to interaction of nanoparticles with the cell membrane^4,36^. Consequently, the measurement of surface pressure-area isotherms of PC in the presence of Si/SiO_2_ QDs using the Langmuir-Blodgett trough, provided for the first time useful insights (Fig. 5) regarding the interaction between this type of nanoparticles and the phospholipid monolayer, as a model system of biomembrane. The effects induced by Si/SiO_2_ QDs on the phospholipid monolayer in the absence, or in the presence, of serum proteins, were schematically illustrated in the Fig. 6, based on the data obtained in our study.

The isotherms of PC showed important changes in the presence of QDs in MEM compared to PC standard alone, most probably due to the intrinsic property of QDs to induce the lipid peroxidation, as it was described by the toxicity mechanism for these nanoparticles^37^. This oxidative injury affected the structure of phospholipids and disturbed their organization creating holes in the monolayer, which resulted in an increase in elasticity. Alternatively, the interaction with QDs could cause the expulsion of PC molecules together with nanoparticles, creating a transient membrane leakage, as it was revealed by hemolysis assay (Fig. 4a). By contrast, QDs covered with protein corona strongly interacted with PC and were incorporated among the lipids increasing the stiffness of monolayer. The phase-contrast images (Fig. 5c) showed that QDs became an integral part of the monolayer, creating complex structures with proteins and lipids. The same effect of incorporation was also described for polyorganosiloxane nanoparticles that interacted with lung surfactant monolayers^38^. This similarity could suggest that protein corona is the key factor in this strong interaction between QDs and phospholipids. Taking into account that it has been previously shown the negatively charged nanoparticle-induced gelation in fluid bilayers by surface reconstruction^39^ and the effect of charged nanoparticles on dipalmitoylphosphatidyl glycerol monolayer^40^, it appears that electrostatic forces and changes in entropy contribute significantly to the interactions at the QDs-bio interface.

The complex characterization of Si/SiO_2_ QDs behavior in a specific biological environment, that was described in the present study, complete the recent researches performed in this domain^41–43^. Using a novel combination of multidisciplinary approaches, the present study revealed the formation of dynamic interactions between QDs and proteins that promoted the formation of QDs-protein corona interface responsible for an enhanced affinity for membrane phospholipids, attenuating the cell membrane damage and QDs toxicity. Therefore, we may conclude that Si/SiO_2_ QDs represent a desirable alternative as compatible biomaterial for further nanomedicine and bioimaging purposes.

## Methods

### Chemicals

Ultrapure Milli-Q water was used in all experiments. Bovine serum albumin (BSA; Cat. No. A7906), Coomassie Brilliant Blue R-250 (Cat. No. 27816), Triton X-100 (Cat. No. T9284), hypericin (Cat. No. H9252), NaCl (Cat. No. 71380) and chloroform (Cat. No. 2432) were obtained from Sigma-Aldrich, USA. Phosphate buffered saline (PBS; Cat. No. 18912-014), fetal bovine serum (FBS; Cat. No. 10270-106), Minimum Essential Medium (MEM; Cat. No. 61100-087) containing Earle’s salts, 2 mM L-glutamine, 1.5 g/l sodium bicarbonate and 1 mM sodium pyruvate, and prestained protein molecular weight marker (Cat. No. LC5925) were purchased from Life Technologies, Scotland. L-α-phosphatidylcholine (PC; Cat. No. 840051) was purchased from Avanti Polar Lipids, USA.

### Nanoparticles

The Si/SiO_2_ QDs with a crystalline silicon core covered by an amorphous SiO_2_ shell were synthesized by pulsed laser ablation. The nanoparticles displayed a mean size between 6 and 8 nm. The synthesis method and the physicochemical characterization of these Si/SiO_2_ QDs were described in detail elsewhere^37,44^. One mg/ml Si/SiO_2_ QDs stock suspension was obtained in ultrapure water, sonicated for 5 min using an ultrasonic processor (UP50H, Hielscher, Germany) and diluted in various media for QDs characterization, sodium dodecyl sulfate polyacrylamide gel electrophoresis (SDS-PAGE) and Langmuir monolayers assays.

### QDs characterization

The characterization of hydrodynamic size and zeta potential of Si/SiO_2_ QDs in ultrapure Milli-Q water, PBS, 20 µg/ml BSA prepared in PBS, 0.9% saline (data not shown; the results were almost similar to those obtained for PBS), MEM without FBS and MEM supplemented with 10% FBS was assessed using a Malvern Nano-ZS instrument (Malvern Instruments, Malvern, UK). A concentration of 100 µg/ml Si/SiO_2_ QDs was incubated with each of these types of media at room temperature for 30 min, 1 h, 3 h, 5 h and 24 h. The measurements were performed in triplicate at 25 °C using the refractive index of 1.52.

### SDS-PAGE

The time evolution of QDs protein corona was assessed by SDS-PAGE. A concentration of 100 µg/ml Si/SiO_2_ QDs was incubated with 20 µg/ml BSA prepared in PBS and with MEM supplemented with 10% FBS at 37 °C for 5 min, 15 min, 30 min, 1 h, 3 h, 5 h, 8 h and 24 h. At the end of each time interval of incubation, the suspensions were centrifuged at 14,000 × *g* for 10 min and washed with PBS three times. The pellets consisting of QDs with adsorbed proteins were mixed with sample loading buffer, all the content was loaded with a Hamilton syringe on a 10% SDS-PAGE and run under reducing conditions. The gels were stained using Coomassie Brilliant Blue G-250. The bands were visualized on a ChemiDoc MP Imaging system (Bio-Rad, USA) and were quantified using the Image Lab 5.0 software (Bio-Rad, USA). BSA (0.125–20 µg protein) and MEM + 10% FBS (0.75–90 µg protein) calibration curves were obtained by SDS-PAGE and these were used in order to calculate the amount of BSA and total protein, respectively, adsorbed on QDs. This amount represents also the quantity of protein loaded on gel which was not measured before the SDS-PAGE by standard spectrophotometric methods (such as Bradford or Lowry protein assays) as these can have multiple disadvantages, including the errors given by the nanoparticle interference and the low protein amount (around 1 µg of protein in the case of BSA-QDs samples). The Bradford reagent and BSA standard were used in the spectrophotometric method in order to quantify the protein concentration of FBS stock solution before starting the incubation experiments.

### Fourier transform infrared spectroscopy (FTIR) measurements

FTIR measurements were performed on a Bruker Tensor 27 spectrometer (Bruker, Germany) in attenuated total reflection (ATR) configuration. Dried samples of BSA, QDs and BSA-QDs suspensions (1 mg/ml Si/SiO_2_ QDs incubated with 10 mg/ml BSA in 1:1 ratio for different periods of time −5 min, 30 min, 1 h, 3 h, 5 h and 24 h) were deposited on the diamond crystal. ATR spectra in the 4000–400 cm^−1^ frequency range, with 4 cm^−1^ resolution, were acquired during a collection time of 1 min. Spectra analysis (including peaks identification, smoothing, and baseline correction) was performed using OPUS software (Bruker, Germany). Deconvolution of amide I bands of BSA and BSA-QDs samples was performed on the baseline corrected spectra passing through the ordinate at 1700 cm^−1^ and 1600 cm^−1^. The location of constituent bands was identified by analyzing the minima on their second derivatives spectra. The Gaussian multiple-peak fitting of amide I bands obtained using the identified locations was performed with OriginPro 2015 using a Levenberg–Marquardt iterative process. Since the use of exact locations of bands identified from the second derivatives spectra does not always lead to a convergent fit, we manually adjusted the locations, introduced new bands or removed bands until the fitting process converged. Secondary structures assignment was performed considering the following frequency ranges:^45^ (i) β-sheet – 1610–1640 cm^−1^; (ii) random coil – 1640–1650 cm^−1^; (iii) α-helix – 1650–1658 cm^−1^; (iv) β-turn – 1660–1700 cm^−1^. The relative percentages of secondary structures were estimated by considering the areas below the corresponding Gaussians.

### Hemolysis assay

Fresh human venous blood was collected into vials with sodium citrate as anticoagulant from healthy volunteers with informed oral consent, under the full institutional ethical approval of the University of Bucharest and following the principles in the Declaration of Helsinki. RBCs isolation and hemolysis assay were performed using the method described by Lu *et al*.^46^. Briefly, the blood was centrifuged at 1,200 × g for 10 min in order to obtain the RBCs which were further washed in 0.9% saline and finally diluted to a concentration 5% RBC (v/v) in saline. The Si/SiO_2_ QDs stock suspensions of 1 mg/ml were prepared in 0.9% saline, serum-free MEM and MEM supplemented with 10% FBS, sonicated for 2 min and incubated at a concentration of 100 µg/ml QDs with the RBC suspension for up to 24 h on a rotating tube shaker at room temperature. In parallel, RBCs incubated with biological media (0.9% saline, serum-free MEM and MEM supplemented with 10% FBS) without QDs and with 0.1% Triton X-100 solution were used as negative and positive control, respectively. At the end of incubation time, the tubes were centrifuged for 5 min and the supernatants were subjected to spectrophotometric analysis at 540 nm by reading the optical density (OD) in order to establish the level of hemoglobin released. The percentage of hemolysis was calculated related to the complete hemolysis induced by Triton X-100, according to the next formula:

1 $$ \% \,{\rm{hemolysis}}=(({{\rm{OD}}}_{{\rm{media}}{\rm{with}}{\rm{QDs}}}-{{\rm{OD}}}_{{\rm{control}}{\rm{media}}})\times 100)/({{\rm{OD}}}_{{\rm{control}}{\rm{Triton}}{\rm{X}}-100}-{{\rm{OD}}}_{{\rm{control}}{\rm{media}}}).$$

### Confocal fluorescence microscopy

Hypericin is a fluorescent extract from St. John’s Worth (*Hypericum perforatum* L.) which is able to bind cell membranes. Thus, it was used to stain the RBCs membranes after the incubation with QDs following a modified method previously described^47^. A 2 mM hypericin stock solution prepared in absolute ethanol was freshly diluted to a final concentration of 10 µM in FBS. Then, 4 µl of RBCs pellets obtained during the hemolysis assay were mixed with an equal volume of diluted hypericin solution on a glass slide and covered by a cover glass. The labeled cell membranes were visualized using a Nikon A1 laser scanning confocal microscope (Tokyo, Japan) with the Apo-TIRF 60×/1.49 NA oil immersion objective. The laser excitation was set at 561 nm and the emission was collected using a 570–620 nm band pass filter.

### Langmuir-Blodgett monolayer assay

A Langmuir-Blodgett trough (NIMA 102 A, NIMA Technologies, UK) was firstly filled with ultrapure water. A lipid monolayer was formed at air-water interface by dropping 20 µl of 0.42 mg/ml PC solution prepared in chloroform using a Hamilton glass syringe obtaining an area per molecule of ∼120 Å^2^. Chloroform was allowed to evaporate during 15 min. Afterwards, the compression of monolayers was initiated with the two movable barriers at a rate of 30 Å^2^ molecule-1 min-1 and ended when the monolayer collapsed. A filter paper Wilhelmy plate was used to monitor the surface pressure levels. In order to assess the effect of Si/SiO_2_ QDs resuspended in serum-free MEM and MEM supplemented with 10% FBS on the lipid monolayer, 100 µg/ml QDs were carefully injected into the aqueous subphase below the interface. After the stabilization of surface pressure for 20 min, the level of surface pressure during compression was recorded as a function of trough area by the NIMA software during compression and further represented as a function of molecular area. All experiments were performed at 25 ± 0.3 °C in triplicate. The analysis of isotherms was performed by calculating the compressional modulus (*Cs*^−1^) for each QDs-lipid monolayer using the following equation:

2 $$C{s}^{-1}=-A(\partial \pi /\partial A),$$

where π is the surface pressure and Å^2^ is the area per molecule. The morphology of PC monolayers collected on clean glass slide was visualized using an Olympus IX71 inverted phase-contrast microscope (Olympus, Tokyo, Japan).

## References

[1] Stan, M. S., Sima, C. & Dinischiotu, A. Silicon quantum dots: from synthesis to bioapplications in *Bioactivity of Engineered* Nanoparticles (eds Yan, B., Zhou, H. & Gardea-Torresdey, J. L.) 339–359 (Springer, 2017).

[2] Xing Y, Rao J (2008). Quantum dot bioconjugates for *in vitro* diagnostics & *in vivo* imaging. Cancer Biomarkers.

[3] Zrazhevskiy P, Sena M, Gao X (2010). Designing multifunctional quantum dots for bioimaging, detection, and drug delivery. Chem. Soc. Rev..

[4] Fadeel B, Feliu N, Vogt C, Abdelmonem AM, Parak WJ (2013). Bridge over troubled waters: understanding the synthetic and biological identities of engineered nanomaterials. Wiley Interdiscip. Rev. Nanomed. Nanobiotechnol..

[5] Slack, S.M. & Horbett, T.A. The Vroman Effect. A Critical Review in *Proteins at Interfaces II. Fundamentals and Applications* (eds. Horbett, T.A. & Brash, J.L.) 112-128 (American Chemical Society, 1995).

[6] Fenoglio I, Fubini B, Ghibaudi EM, Turci F (2011). Multiple aspects of the interaction of biomacromolecules with inorganic surfaces. Adv. Drug Deliv. Rev..

[7] Lesniak A (2012). Effects of the presence or absence of a protein corona on silica nanoparticle uptake and impact on cells. ACS Nano.

[8] Wu XY, Narsimhan G (2008). Effect of surface concentration on secondary and tertiary conformational changes of lysozyme adsorbed on silica nanoparticles. Biochim. Biophys. Acta.

[9] Shang W (2009). Cytochrome C on silica nanoparticles: influence of nanoparticle size on protein structure, stability, and activity. Small.

[10] Deng ZJ, Liang M, Monteiro M, Toth I, Minchin RF (2011). Nanoparticle-induced unfolding of fibrinogen promotes Mac-1 receptor activation and inflammation. Nat. Nanotechnol..

[11] Sánchez-Pérez JA, Gallardo-Moreno AM, González-Martín ML, Vadillo-Rodríguez V (2015). BSA adsorption onto nanospheres: Influence of surface curvature as probed by electrophoretic light scattering and UV/vis spectroscopy. Appl. Surf. Sci..

[12] Casals E, Pfaller T, Duschl A, Oostingh GJ, Puntes V (2010). Time evolution of the nanoparticle protein corona. ACS Nano.

[13] Strojan K (2017). Dispersion of nanoparticles in different media importantly determines the composition of their protein corona. PLoS ONE.

[14] Roh J (2013). Dispersion stability of citrate- and PVP-AgNPs in biological media for cytotoxicity test. Korean J. Chem. Eng..

[15] Mansour N, Momeni A, Karimzadeh R, Amini M (2012). Blue-green luminescent silicon nanocrystals fabricated by nanosecond pulsed laser ablation in dimethyl sulfoxide. Optical Materials Express.

[16] Givens BE, Xu Z, Fiegel J, Grassian VH (2017). Bovine serum albumin adsorption on SiO2 and TiO2 nanoparticle surfaces at circumneutral and acidic pH: A tale of two nano-bio surface interactions. J. Colloid Interface Sci..

[17] Qing H, Yanlin H, Fenlin S, Zuyi T (1996). Effects of pH and metal ions on the conformation of bovine serum albumin in aqueous solution. An attenuated total reflection (ATR) FTIR spectroscopic study. Spectrochim. Acta. A. Mol. Biomol. Spectrosc..

[18] Podila R, Chen R, Ke PC, Brown JM, Rao AM (2012). Effects of surface functional groups on the formation of nanoparticle-protein corona. Appl. Phys. Lett..

[19] ASTM F756-00, Standard Practice for Assessment of Hemolytic Properties of Material (ASTM International, 2004).

[20] Valton E, Amblard C, Wawrzyniak I, Penault-Llorca F, Bamdad M (2013). P-gp expression in brown trout erythrocytes: evidence of a detoxification mechanism in fish erythrocytes. Sci. Rep..

[21] Boulos SP (2013). Nanoparticle-protein interactions: a thermodynamic and kinetic study of the adsorption of bovine serum albumin to gold nanoparticle surfaces. Langmuir.

[22] Tenzer S (2013). Rapid formation of plasma protein corona critically affects nanoparticle pathophysiology. Nat. Nanotechnol..

[23] Vroman L, Adams AL, Fischer GC, Munoz PC (1980). Interaction of high molecular weight kininogen, factor XII, and fibrinogen in plasma at interfaces. Blood.

[24] Corbo C (2016). The impact of nanoparticle protein corona on cytotoxicity, immunotoxicity and target drug delivery. Nanomedicine (Lond).

[25] Sakulkhu U, Mahmoudi M, Maurizi L, Salaklang J, Hofmann H (2014). Protein corona composition of superparamagnetic iron oxide nanoparticles with various physico-chemical properties and coatings. Sci Rep..

[26] Rausch K, Reuter A, Fischer K, Schmidt M (2010). Evaluation of nanoparticle aggregation in human blood serum. Biomacromolecules.

[27] Safi M, Courtois J, Seigneuret M, Conjeaud H, Berret JF (2011). The effects of aggregation and protein corona on the cellular internalization of iron oxide nanoparticles. Biomaterials.

[28] Chatterjee S, Mukherjee TK (2014). Spectroscopic investigation of interaction between bovine serum albumin and amine-functionalized silicon quantum dots. Phys. Chem. Chem. Phys..

[29] Bardajee GE, Hooshyar Z (2016). Interaction of a novel starch-capped CdS quantum dots with human serum albumin and bovine serum albumin. Starch/Stärke.

[30] Rajeshwari A (2014). Spectroscopic studies on the interaction of bovine serum albumin with Al_2_O_3_ nanoparticles. J. Lumin..

[31] Abrosimova KV, Shulenina OV, Paston SV (2016). FTIR study of secondary structure of bovine serum albumin and ovalbumin. J. Phys.: Conf. Ser..

[32] Joglekar M, Roggers RA, Zhao Y, Trewyn BG (2013). Interaction effects of mesoporous silica nanoparticles with different morphologies on human red blood cells. RSC Adv..

[33] Adewoyin AS, Nwogoh B (2014). Peripheral blood film – a review. Ann. Ibd. Pg. Med..

[34] Yoo D, Lessin LS (1992). Drug-associated “bite cell” hemolytic anemia. Am. J. Med..

[35] Kutwin M (2014). Structural damage of chicken red blood cells exposed to platinum nanoparticles and cisplatin. Nanoscale Res. Lett..

[36] Mahmoudi M (2014). Interaction of stable colloidal nanoparticles with cellular membranes. Biotechnol. Adv..

[37] Stan MS (2014). Si/SiO2 quantum dots cause cytotoxicity in lung cells through redox homeostasis imbalance. Chem. Biol. Interact..

[38] Harishchandra RK, Saleem M, Galla HJ (2010). Nanoparticle interaction with model lung surfactant monolayers. J. R. Soc. Interface.

[39] Wang B, Zhang LF, Bae SC, Granick S (2008). Nanoparticle-induced surface reconstruction of phospholipid membranes. Proc. Natl. Acad. Sci. USA.

[40] Torrano AA, Pereira AS, Oliveira ON, Barros-Timmons A (2013). Probing the interaction of oppositely charged gold nanoparticles with DPPG and DPPC Langmuir monolayers as cell membrane models. Colloids Surf. B: Biointerfaces.

[41] Stan MS, Sima C, Cinteza LO, Dinischiotu A (2015). Silicon-based quantum dots induce inflammation in human lung cells and disrupt extracellular matrix homeostasis. FEBS J..

[42] Ostrovska L (2016). The impact of doped silicon quantum dots on human osteoblasts. RSC Adv..

[43] Wang, H. *et al*. The nature of a hard protein corona forming on quantum dots exposed to human blood serum. *Small*, 10.1002/smll.201602283 (2016). 10.1002/smll.201602283 27606563

[44] Petrache SN (2012). Structural and oxidative changes in the kidney of crucian carp induced by silicon-based quantum dots. Int. J. Mol. Sci..

[45] Surewicz WK, Mantsch HH (1988). New insight into protein secondary structure from resolution-enhanced infrared-spectra. Biochim. Biophys. Acta.

[46] Lu S (2009). Efficacy of simple short-term *in vitro* assays for predicting the potential of metal oxide nanoparticles to cause pulmonary inflammation. Environ. Health Perspect..

[47] Constantinescu AA (2010). Studying the aging of banked erythrocytes using a fluorescence marker, hypericin. Annals of the “Alexandru Ioan Cuza” University Sect.II a. Genetics and Molecular Biology.

